# Anti-MRSA Activity of Actinomycin X_2_ and Collismycin A Produced by *Streptomyces globisporus* WA5-2-37 From the Intestinal Tract of American Cockroach (*Periplaneta americana*)

**DOI:** 10.3389/fmicb.2020.00555

**Published:** 2020-04-07

**Authors:** Zhiyu Chen, Peiyu Ou, Lingyan Liu, Xiaobao Jin

**Affiliations:** ^1^School of Life Sciences and Biopharmaceutics, Guangdong Pharmaceutical University, Guangzhou, China; ^2^Guangdong Provincial Key Laboratory of Pharmaceutical Bioactive Substances, Guangdong Pharmaceutical University, Guangzhou, China

**Keywords:** methicillin-resistant *Staphylococcus aureus*, *Streptomyces globisporus*, *Periplaneta americana*, actinomycin X_2_, collismycin A

## Abstract

Methicillin-resistant *Staphylococcus aureus* (MRSA) is recognized as one of the serious pathogen that causes acquired infections worldwide. Its emerging need to discover novel, safe and potent anti-MRSA drugs. In this study, primary screening by anti-MRSA activity assay found one strain WA5-2-37 isolated from the intestinal tract of *Periplaneta americana*, exhibited great activity against MRSA ATCC 43300. The strain WA5-2-37 produced actinomycin X_2_ and collismycin A which showed strong inhibition of MRSA with minimum inhibitory concentration (MIC) values of 0.25 and 8 μg/mL. The structures of the pure compounds were elucidated by analysis of mass spectrometry (MS), ^1^H and ^13^C nuclear magnetic resonance (NMR). The strain WA5-2-37 was considered as *Streptomyces globisporus* on the basis of morphological characteristics, genotypic data, and phylogenetic analysis. This is the first reported naturally occurring strain of *S. globisporus* isolated from the intestinal tract of *P. americana*, whereas it has almost been found from plants, marine, and soil previously. Moreover, *S. globisporus* has not been reported to produce any anti-MRSA substances previously, such as actinomycin X_2_ and collismycin A. In conclusion, the insect-derived strain of *S. globisporus* WA5-2-37 was considered of great potential as a new strain of producing actinomycin X_2_, collismycin A or other anti-MRSA compounds.

## Introduction

*Streptomyces globisporus* has been isolated from soil, plants, and so on (Jizba et al., 1991; Li et al., 2012), but rarely from insects. Previous literatures also showed that *S. globisporus* was able to produce a wide variety of metabolites with antibacterial, antifungal, and antitumor activities, such as globimycin, radamycin, methylsulfomycin, etc. (Li X. et al., 2016; Kaweewan et al., 2017; Kemung et al., 2018). Therefore, *S. globisporus* are considered as an economically and biotechnologically useful microorganism due to its diversity and proven ability to produce bioactive compounds. However, it has not reported that *S. globisporus* could produce any bioactive compounds with anti-MRSA activity.

Cockroach is one of the most diverse and abundant pests all over the world (Hassan, 2017). *Periplaneta americana*, a largest species of common cockroach, has been extensively studied for its certain special substances and defense mechanisms to resist environmental threats (Li N. et al., 2016). Moreover, *P. americana* is a well-known medicinal insect in China with excellent therapeutic effects on wound repair and ulcer (Owen and Bouquillon, 1992; Song et al., 2017). But only few chemical investigations were reported on the title insect, especially the metabolites of the symbiotic microorganism of *P. americana*.

In this study, 159 strains were isolated from the intestinal tract of *P. americana* and 1 *S. globisporus* strain, WA5-2-37, showed great activity against MRSA ATCC 43300. Herein, this study aimed to determine the identity of the anti-MRSA compounds produced by *S. globisporus* strain WA5-2-37 and described the anti-MRSA action of the compounds.

## Materials and Methods

### Microorganisms, Chemicals, and Media

The strain of bacteria was tested: MRSA ATCC 43300 which were obtained from Guangdong culture collection center. The genomic DNA isolation kit was purchased from Sangon Biotech. Microbiological media were purchased from Guangdong Huankai Microbial Sci. & Tech. Co., Ltd. High-performance liquid chromatography (HPLC) grade methanol was purchased from Honeywell. Solvents used for extraction and column chromatography were of analytical grade which were obtained from Guangdong Guanghua Sci-Tech Co., Ltd.

### Primary Anti-MRSA Activity Assay

Primary screening for anti-MRSA activity was performed by growing cultures on Luria-Bertan (LB) agar plates swabbed with the MRSA ATCC 43300 suspension (1–5 × 10^4^ CFU/mL). 90 μL of the ethyl acetate extract obtained from each tested strain was added to an oxford cup on the LB agar plates and the incubated at 37°C for 12 h (Song et al., 2018). Vancomycin was used as positive control with the concentration of 100 μg/mL and the experiment was performed in triplicate. The diameter of the inhibition zone was recorded the anti-MRSA activity of each tested strain expressed millimeter.

### Isolation and Identification of *S. globisporus* Strain WA5-2-37

The *P. americana* insects in this study were reared and provided by Guangdong Provincial Center for Disease Control and Prevention. The insects were firstly paralyzed at 4°C and washed alternately with water and 75% ethanol 3 times before dissection. Then the intestinal tract of *P. Americana* was dissected and removed with a sterile scalpel. The intestinal tract samples were grinded with sterile water and diluted to 1 × 10^–3^ g/mL, 2 × 10^–3^ g/mL, and 4 × 10^–3^ g/mL. Then the samples were coated on plate medium and cultured at 28°C for 7 days. Finally, the colony was purified and re-cultured, and a single colony was obtained according to the method of reference (Cook et al., 2007; Wang et al., 2014).

*Streptomyces globisporus* strain WA5-2-37 was inoculated on Gause’s synthetic agar no. 1 plates and incubated at 28°C for 4 days, during which time the culture had entered the logarithmic growth phase. The cultural and morphological characteristics of *S. globisporus* strain WA5-2-37 on Gause’s synthetic agar were tentatively identified based on the published descriptions of various Streptomyces species (Shirling and Gottlieb, 1968a, b, 1969, 1972). Whole genomic DNA of *S. globisporus* strain WA5-2-37 was extracted using the GenElute Bacterial Genomic DNA Kit (Sigma) according to manufacturer’s instructions. The 16S rDNA gene was then amplified by PCR, using universal bacterial primer set 27F (5′-AGA GTT TGA TCM TGG CTC AG-3′) and 1492R (5′-TAC GGC TAC CTT GTT ACG ACT T-3′) (Hamedi et al., 2013). The PCR was performed in a total volume of 25 μL containing 1 μL DNA template, 1 μL upstream primer (Invitrogen), 1 μL downstream primer (Invitrogen), 12.5 μL PCR Premix (Invitrogen) and 9.5 μL ddH_2_O. The PCR cycle consisted of a 94°C pre-denaturation for 4 min, 35 cycles of three steps (94°C denaturation for 30 s, 55°C for annealing for 30 s, 72°C extension for 30 s), and 72°C post-cycle extension for 5 min. After that, PCR products were verified by electrophoresis on a 1.5% agarose gel and was sequenced by Beijing Genomics Institute (Beijing, China). Sequences analysis of 16S rDNA was then conducted using BLAST hosted by National Center for Biotechnology Information (NCBI) website^1^. Sequences were aligned with Clustal software and MEGA 7.0 was used to carry out a phylogenetic tree of the alignment using the maximum likelihood method (Kumar et al., 2008; Tamura et al., 2011).

### Fermentation, Extraction, and Isolation

Single colonies of *S. globisporus* strain WA5-2-37 were inoculated into a 250 mL flask containing 100 mL of ISP-1 medium as seed and then incubated on a rotary shaker at 28°C and 150 rpm for 2 days, which was used as seed culture. From this, 5% of this seed was transferred to 500 mL flask containing 300 mL of ISP-2 medium as production flasks and incubated at 28°C and 150 rpm for 7 days. The total 40 L of fermentation were extracted with an equal volume of ethyl acetate and 12.0 g red crude extract was obtained, and the crude extract was subjected to a bioactivity guided fractionation (Esposito et al., 2016). The crude extract was loaded onto a silica gel column using gradient elution with an increasing polarity of ethyl acetate (1–100%) and hexane to given 12 major fractions (A–L). Every fraction was used for testing for the active component against MRSA ATCC 43300. The active fractions were further purified by semi-preparative HPLC using a YMC-Pack ODS-AQ C18 column (10 mm × 250 mm, YMC, Japan) in a methanol solvent system collection of fractions.

### Spectroscopic Analysis

The active and pure fraction was analyzed by analytical HPLC using a YMC-Pack ODS-AQ C18 column (4.5 mm × 250 mm, YMC, Japan) with a photodiode array detector (PAD). The proton and carbon nuclear magnetic resonance (NMR) spectra were recorded at 600 MHz using a Brucker AVANCE III 600M spectrometer (Brucker) with tetramethylsilane (TMS) as an internal reference and dimethyl sulfoxide (DMSO) as solvent.

### Minimum Inhibitory Concentration Testing

The minimum inhibitory concentration (MIC) for purified compounds against the MRSA were determined by the tube-dilution method using individually pack-aged, flat bottomed, 96-well microtiter plates (Liu et al., 2017). The tested bacterial strain was cultivated in Mueller-Hinton Broth at 37°C until the cellular density reached approximately 1 ∼ 5 × 10^5^ CFU/mL. Each of the tested compounds and drugs were dissolved in DMSO and then diluted with sterile broth by the twofold dilution method. The final concentrations of each sample in the wells were 512, 256, 128, 64, 32, 16, 8, 4, 2, 1, 0.5, 0.25, and 0.125 μg/mL. Vancomycin was used as positive control and ampicillin was used as a control to prove that the tested bacteria were MRSA. A serial dilution of compounds were performed in the microplates and incubated at 37°C for 12 h. All the experiment was performed in triplicate and the last tube with no growth of microorganism was recorded as the MIC value expressed in μg/mL.

### Scanning Electron Microscopy Analysis

The morphological changes of the tested MRSA were determined by scanning electron microscopy (SEM). The tested bacterial suspension (1 ∼ 5 × 10^5^ CFU/mL) were treated with 4× the MIC of the compound for 12 h at 37°C. Cells were then placed onto a glass coverslip and fixed with 2.5% glutaraldehyde at 4°C overnight. Fixed samples were washed with 1× phosphate buffered saline (PBS) three times for 20 min each and dehydrated in increasing concentrations of ethanol (20, 40, 60, 80, and 100%). The coverslips were finally dried then analyzed by Quanta FEG 200 FESEM at an accelerating voltage of 2–19 kV under standard operating conditions.

### GenBank Submission

The partial sequence of the 16S rDNA gene of isolate WA5-2-37 was submitted to NCBI GenBank under accession number MH304280.

## Results

### Screening of the Strains With Anti-MRSA Activity and Identification of Strain WA5-2-37

**FIGURE 1:**
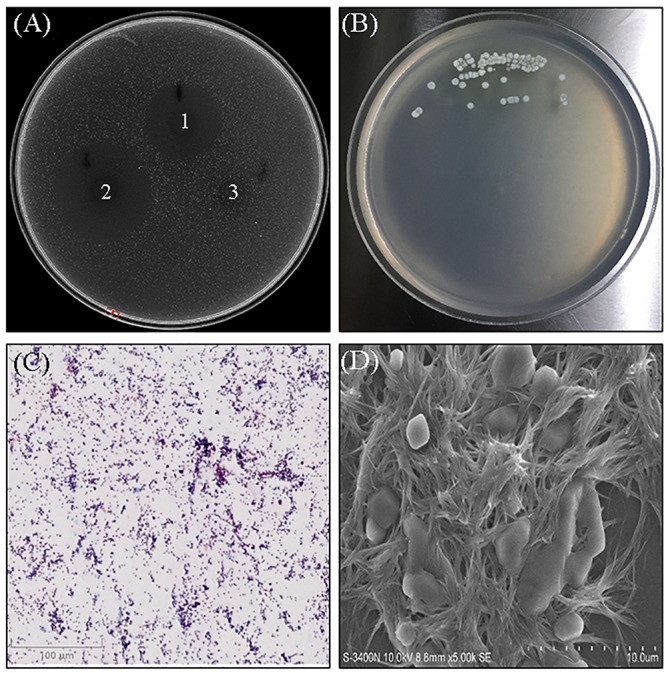
The anti-MRSA activity, morphological characteristics of strain WA5-2-37. **(A)** The anti-MRSA activity of WA5-2-37, 1–3 refers to sample, positive control and blank control. The concentrations of sample and positive control were both 100 μg/mL and the experiment was performed in triplicate. **(B)** Culture on Gause’s synthetic agar no. 1. **(C)** Observation of the culture under optical microscopy following Gram staining (100×). **(D)** Observation of the culture by scanning electron microscopy (5,000×).

**FIGURE 2:**
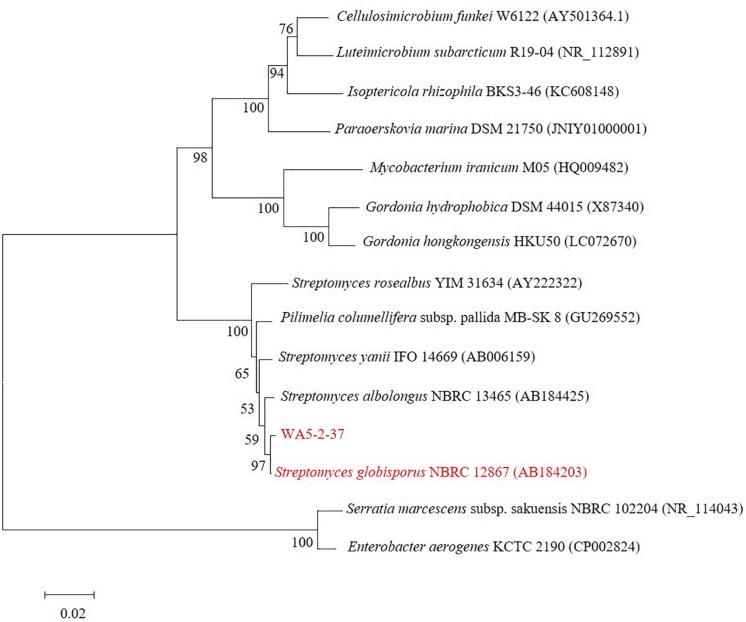
The phylogenetic tree of strain WA5-2-37. Maximum-likelihood phylogenetic tree based on 16S rDNA gene sequences showing the positions of strain WA5-2-37, the type strains of other *Streptomyces* and representatives of some other related taxa. Only bootstrap values (expressed as percentages of 1,000 replications) greater than 50% are shown at branching points. Bar, 0.02 substitutions per nucleotide position.

The screening for anti-MRSA activity showed the strain WA5-2-37 had great activity against MRSA ATCC 43300 (Figure 1A). Based on the cultural and morphological characteristics showed in Figures 1B–D, strain WA5-2-37 belonged to genus *Streptomyces*. The strain appeared as leathery colonies with abundant spores (powder) on the Gause’s synthetic agar no. 1 plates. The Gram staining result showed strain WA5-2-37 was the Gram-positive bacterium. The morphological characteristics were observed under SEM and the result showed that the spore of strain WA5-2-37 were found to be oval to round in shape. The phylogenetic analyses (Figure 2) based on 16S DNA sequences and the taxonomic position of strain WA5-2-37 in the phylogenetic tree indicated that strain WA5-2-37 was most similar to *S. globisporus* NBRC 12867 (AB184203). Based on these result, strain WA5-2-37 was preliminary confirmed as *S. globisporus*.

### Isolation and Purification of the Anti-MRSA Compounds

Strain WA5-2-37 was cultured by the shake-flask method, after which the culture broth (40 L) was then centrifuged at 4000 rpm at 4°C cell-free supernatants were extracted with an equal volume of ethyl acetate. The organic layer was then concentrated *in vacuo* using a rotary evaporator. After that, the crude extract (12.0 g) of strain WA5-2-37 was subjected to bioactivity guided isolation and purification. Twelve different major fractions (A–L) were obtained after eluting by gradient elution with an increasing polarity of ethyl acetate (1–100%) and hexane. Each fraction was then concentrated and tested for activity against MRSA. The fraction A and D showed the most potent activity against MRSA ATCC 43300. The fraction A were separated further on Spehadex LH-20 with a solvent gradient of 100% methanol and purified by semi-preparative HPLC with a solvent gradient of 80% methanol. The fraction D were purified by semi-preparative HPLC with a solvent gradient of 70% methanol. There two pure compounds were obtained finally.

### Spectroscopic Analysis

**FIGURE 3:**
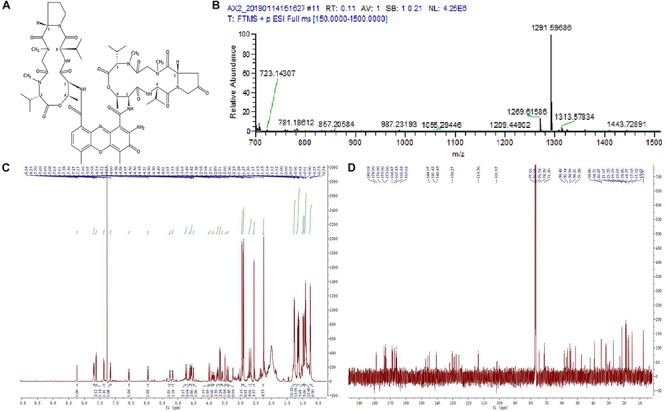
Structure identification of the purified compound 1. **(A)** Molecular structure of the compound. **(B)** Mass spectrometry. **(C)** Proton (^1^H) nuclear magnetic resonance. **(D)** Carbon (^13^C) nuclear magnetic resonance spectrum of the compound 1 in DMOS-6*d*.

**FIGURE 4:**
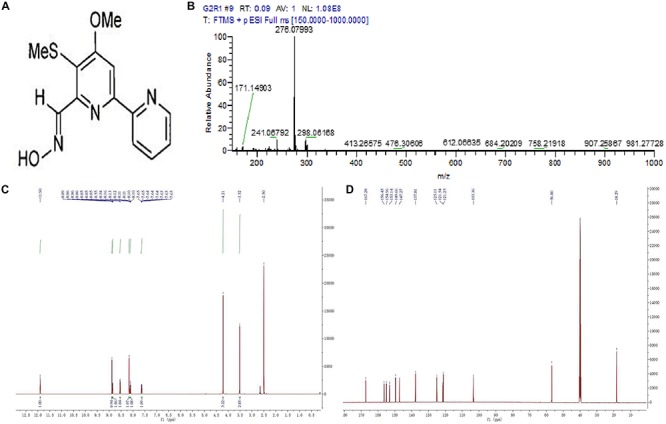
Structure identification of the purified compound 2. **(A)** Molecular structure of the compound. **(B)** Mass spectrometry. **(C)** Proton (^1^H) nuclear magnetic resonance. **(D)** Carbon (^13^C) nuclear magnetic resonance spectrum of the compound 2 in DMOS-6*d*.

The structures of two pure compounds were elucidated by high resolution electrospray ionization mass spectrometry (HRESIMS) and NMR analysis as well as by comparison with the previously reported MS and NMR data. HRESIMS of pure compounds showed molecular ion peaks at *m*/*z* 1291.59686 [M + Na]^+^ for compound 1 (Figure 3B) and 276.07993 [M + H]^+^ for compound 2 (Figure 4B). The ^1^H and ^13^C NMR spectra of pure compounds showed a great similarity to the previously reported data (Figures 3C,D, 4C,D). Therefore, the structures of compounds 1 and 2 were confirmed to be actinomycin X_2_ and collismycin A (Figures 3A, 4A).

### Anti-MRSA Activity of the Isolated Compounds

**FIGURE 5:**
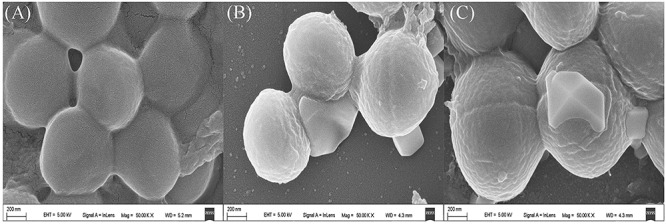
Scanning electron microscopy (SEM) images of pure compounds treated and untreated MRSA (50,000×). **(A)** Untreated MRSA. **(B)** Actinomycin X_2_ treated MRSA. **(C)** Collismycin A treated MRSA.

As the results showed in Table 1, compounds 1 and 2 showed significant activity against MRSA ATCC 43300 with MIC values of 0.25 and 8 μg/mL, which was higher than vancomycin (0.125 μg/mL). Moreover, ampicillin showed no inhibitory activity against MRSA, indicating that the strain was MRSA. The SEM analysis showed the cell membrane of MRSA were destroyed after treating actinomycin X_2_ and collismycin A (Figure 5).

**TABLE 1:** The anti-MRSA activity of compounds isolated from strain WA5-2-37 (MIC, μg/ml, *n* = 3).

**Tested strain**	**Compound 1**	**Compound 2**	**Vancomycin**	**Ampicillin**
Methicillin-resistant *Staphylococcus aureus* ATCC 43300	0.25	8	0.125	–

*–, no activity was detected.*

## Discussion

Methicillin-resistant *S. aureus* is recognized as one of the pathogens that causes hospital- and community-acquired infections worldwide (Sit et al., 2017). MRSA is highly prevalent in hospitals worldwide, especially in Asia (Stefani et al., 2012). Moreover, the outcome of invasive MRSA infection is poor with high 90-day mortality rates from bacteremia (Nickerson et al., 2009; Bal et al., 2017). Therefore, there is an emerging demand for discovery of novel, safe and potent bioactive compounds with anti-MRSA activity (Uchida et al., 2014). Therefore, more and more research efforts have focused on the exploration of microorganism to discover bioactive secondary metabolites (Gos et al., 2017; Sadaf et al., 2018; Lee et al., 2018). But with decreased of the identification of natural products from traditional sources, scientists have turned their eyes to the microorganism from new sources such as insects (Beemelmanns et al., 2017; Ma et al., 2017). Insects, as the most abundant species in nature, can adapt and live in a variety of environments. Moreover, insects contain many types of symbiotic microorganisms in their bodies that produce a variety of bioactive substances (Beemelmanns et al., 2016; Angela, 2018). Therefore, the microbes isolated from insects can be a new source to identify bioactive substances.

In this study, we carried out taxonomic identification of strain WA5-2-37 from the intestinal tract of *P. americana* and isolated and characterized the bioactive metabolites produced by this strain. Strain WA5-2-37 was identified as *S. globisporus* based on morphological characteristics and molecular identification. WA5-2-37 is the first reported strain of *S. globisporus* found in the intestinal tract of *P. americana*. To the best of our knowledge, *S. globisporus* was reported capable of producing a variety of bioactive substances (Chen et al., 2010; Seo et al., 2001), whereas strain WA5-2-37 is the first reported naturally occurring strain of *S. globisporus* with anti-MRSA activity. Without any optimization of the culture conditions, strain WA5-2-37 showed better activity against MRSA ATCC 43300 than other strains in this study. Therefore, optimization of the culture conditions and gene regulation was expected to be done to exploit the full potential for discovery of biologically active substances of this strain.

Actinomycin X_2_ and collismycin A are potent bioactive substances that can be used in the treatment of several human diseases, such as cancer, bacterial infection, and so on (Reich, 1963; Chen et al., 2012). Both of these two compounds can be produce by many *Streptomyces* sp. (Xiong et al., 2008; Kawatani et al., 2016), but it is the first reported that *S. globisporus* can produce actinomycin X_2_ and collismycin A. Moreover, the current studies showed that collismycin A was almost discovered from plants, marine, or soil sources, but never found in insects (Vior et al., 2014; Lee et al., 2017). Herein, this literature is the first reported that a *S. globisporus* isolated from the intestinal tract of *P. americana* with the ability to produce actinomycin X_2_ and collismycin A. In this study, actinomycin X_2_ and collismycin A showed great activity against MRSA ATCC 43300 with the MIC values of 0.25 and 8 μg/mL, and both of them were harmful to the cell membrane of MRSA. However, the specific mechanisms of the anti-MRSA of actinomycin X_2_ and collismycin A were unclear and need to be studied in further research.

## Data Availability Statement

The datasets generated for this study can be found in the NCBI GenBank under accession number MH304280.

## Author Contributions

XJ and ZC conceived and designed the experiments and wrote the manuscript. ZC, PO, and LL performed the experiments and analyzed the data. All authors read and approved the manuscript.

## Conflict of Interest

The authors declare that the research was conducted in the absence of any commercial or financial relationships that could be construed as a potential conflict of interest.

## Footnotes

1 https://www.ncbi.nlm.nih.gov/
